# Integrative multi-omics approach using random forest and artificial neural network models for early diagnosis and immune infiltration characterization in ischemic stroke

**DOI:** 10.3389/fneur.2024.1475582

**Published:** 2024-12-04

**Authors:** Ling Lin, Chunmao Guo, Hanna Jin, Haixiong Huang, Fan Luo, Ying Wang, Dongqi Li, Yuanxin Zhang, Yuqian Xu, Chanyan Zhu, Fengshan Zeng, Huahua He, Jie Chen, Wei Zhang, Wenlin Yu

**Affiliations:** ^1^Department of Neurology, Huizhou Hospital of Guangzhou University of Chinese Medicine (Huizhou Hospital of Traditional Chinese Medicine), Huizhou, Guangdong, China; ^2^Clinical Laboratory, Huizhou Hospital of Guangzhou University of Chinese Medicine (Huizhou Hospital of Traditional Chinese Medicine), Huizhou, Guangdong, China; ^3^Department of Neurology, Shaanxi Provincial Hospital of Chinese Medicine, Xi’an, Shaanxi, China; ^4^Department of Geriatrics, Xi’an Baoshi Flower Changqing Hospital, Xi’an, Shaanxi, China; ^5^Institute of Metabolic Diseases, Guang’anmen Hospital, China Academy of Chinese Medical Sciences, Beijing, China; ^6^School of Basic Medicine, Gansu University of Chinese Medicine, Lanzhou, Gansu, China

**Keywords:** ischemic stroke, differentially expressed genes, random forest, artificial neural network, diagnosis model

## Abstract

**Background:**

Ischemic stroke (IS) is a significant global health issue, causing high rates of morbidity, mortality, and disability. Since conventional Diagnosis methods for IS have several shortcomings. It is critical to create new Diagnosis models in order to enhance existing Diagnosis approaches.

**Methods:**

We utilized gene expression data from the Gene Expression Omnibus (GEO) databases GSE16561 and GSE22255 to identify differentially expressed genes (DEGs) associated with IS. DEGs analysis using the Limma package, as well as GO and KEGG enrichment analyses, were performed. Furthermore, PPI networks were constructed using DEGs from the String database, and Random Forest models were utilized to screen key DEGs. Additionally, an artificial neural network model was developed for IS classification. Use the GSE58294 dataset to evaluate the effectiveness of the scoring model on healthy controls and ischemic stroke samples. The effectiveness of the scoring model was evaluated through AUC analysis, and CIBERSORT analysis was conducted to estimate the immune landscape and explore the correlation between gene expression and immune cell infiltration.

**Results:**

A total of 26 significant DEGs associated with IS were identified. Metascape analysis revealed enriched biological processes and pathways related to IS. 10 key DEGs (ARG1, DUSP1, F13A1, NFIL3, CCR7, ADM, PTGS2, ID3, FAIM3, HLA-DQB1) were selected using Random Forest and artificial neural network models. The area under the ROC curve (AUC) for the IS classification model was found to be near 1, indicating its high accuracy. Additionally, the analysis of the immune landscape demonstrated elevated immune-related networks in IS patients compared to healthy controls.

**Conclusion:**

The study uncovers the involvement of specific genes and immune cells in the pathogenesis of IS, suggesting their importance in understanding and potentially targeting the disease.

## 1 Introduction

Ischemic stroke (IS) is a common cerebrovascular disease characterized by impaired blood supply to the brain, leading to ischemia and hypoxia in brain tissue and resulting in conditions such as brain tissue necrosis (1). Local blood supply disorders in the brain can cause significant neurological damage, affecting speech, movement, balance, and swallowing. Severe brain injury, long-term disability, or even death can occur as a result (2). This leads to prolonged hospital stays, increased medical expenses, and substantial consumption of medical resources, creating a significant economic burden on global healthcare systems. Worldwide, approximately 9.77% of strokes occur in individuals under the age of 35. Additionally, statistics show that about 1 in every 4 individuals over the age of 25 will experience a stroke in their lifetime, indicating a gradual increase in stroke incidence (3).

IS imposes significant psychological and economic burdens on patients, families, and society. It is associated with high morbidity, disability, mortality, and recurrence rates. Therefore, early diagnosis and effective emergency treatment are crucial in reducing the risk of disability and mortality. The key to effective treatment lies in timely restoration of blocked blood vessels and blood supply to save brain tissue from necrosis. Currently, the recommended methods for early blood flow restoration in IS include intravenous thrombolysis and endovascular therapy. Intravenous thrombolysis, in particular, is the preferred treatment option for patients within the time window (4, 5). Recombinant tissue Plasmin activator (rt-PA) is considered the most effective drug for clinical treatment, and its efficacy and safety have been studied in various clinical trials since 1996. Over time, the time window for intravenous thrombolysis has been gradually expanded from the initial 3 h to 4.5 h or even 6 h (6, 7).

**Figure 1:**
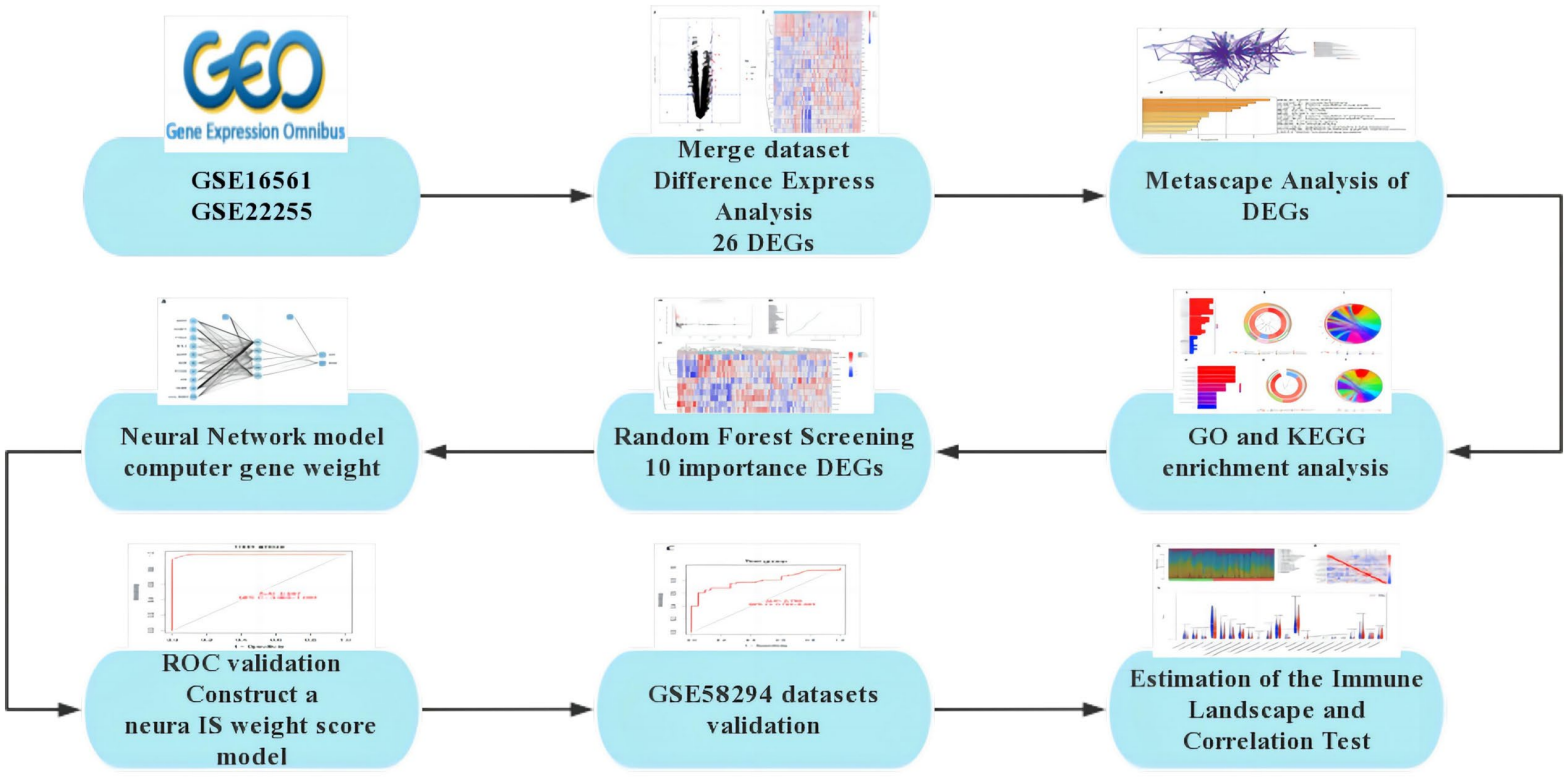
Flowchart.

The severity of neurological deficit in patients with IS can be assessed using the National Institutes of Health Stroke Scale (NIHSS). Studies have shown that the NIHSS score can independently predict the clinical prognosis of IS patients (8). During the first 24 h after onset, the NIHSS score changes dynamically as the disease progresses or is treated. The relationship between the degree of neurological deficit and long-term functional outcome becomes stronger after the initial few hours and then levels off (9). Early diagnosis and treatment of IS can help predict the natural course of the disease, reverse disease progression, improve prognosis, and reduce the incidence of complications such as IS. Therefore, there is an urgent need to supplement existing diagnostic measures and develop new diagnostic models. The rapid advancement of second-generation sequencing technology in recent years has provided a foundation for the discovery of several disease-related genes. In this study, we conducted a search in the GEO database to identify genes that showed differential expression between IS and healthy control samples. Based on these data, we employed Random Forest to discover important genes expressed in IS. Subsequently, we utilized this data to construct an early diagnosis model of IS using the Artificial Neural Network method (Figure 1).

## 2 Materials and methods

### 2.1 Data downloading and analysis

DEGs were identified using the Gene Expression Omnibus (GEO).^1^ The screening criteria included the expression patterns and clinical phenotypic data from the GSE16561, GSE22255, and GSE58294 microarray datasets, which were retrieved using the query tool and presented in Table 1. Annotation data for appropriate platform microarray probes were collected from the GEO database. During the translation of ChIP probe IDs and gene symbols, multiple probes matching a single gene symbol were found. In such cases, the gene expression level was determined based on the average expression level of the probes.

**Table 1:** Data download.

ID	GSE number	Data type	Samples	Source type	Group
1	GSE16561	Illumina HumanRef-8 v3.0 expression beadchip	39 ischemic stroke patients24 healthy control	peripheral whole blood RNA	Discovery cohort
2	GSE22255	[HG-U133_Plus_2] Affymetrix Human Genome U133 Plus 2.0 Array	20 ischemic stroke20 sex- and age-matched controls	Blood genomic expression profile	Discovery cohort
3	GSE58294	[HG-U133_Plus_2] Affymetrix Human Genome U133 Plus 2.0 Array	69 cardioembolic stroke23 control	Blood	Validation cohort

When selecting the datasets for our study, we adhered to a stringent set of criteria to ensure the quality and relevance of the data. We prioritized datasets directly associated with IS and ensured coverage of diverse types of IS patients and control groups to obtain a comprehensive research perspective. We chose three datasets, namely GSE16561, GSE22255, and GSE58294, each containing IS patient and control samples from different experimental platforms. In terms of data preprocessing, we standardized the raw gene expression data using relevant software packages in R to eliminate technical variations. Differential gene analysis using the Limma R package identified the DEGs associated with IS. To enhance statistical power, we combined the data from GSE16561 and GSE22255 and validated the results using the GSE58294 dataset. This systematic preprocessing ensured the reliability of the selected datasets, laying a solid foundation for subsequent bioinformatics analyses.

The fundamental principle behind selecting the three datasets, GSE16561, GSE22255, and GSE58294, is based on the critical information they provide, contributing to the study of gene expression and pathological mechanisms in acute ischemic stroke. The purpose of GSE16561 is to identify a set of genes for the diagnosis of acute ischemic stroke through the analysis of gene expression in peripheral whole blood, providing insights into the biological pathways involved in the human response to acute ischemic stroke. GSE22255 aims to understand the etiology of stroke by analyzing gene expression profiles to better comprehend the complexity of this disease, which has unclear pathogenesis involving environmental and genetic factors. GSE58294 collected blood samples from cardioembolic stroke subjects and controls, exploring the molecular mechanisms of this specific type of stroke through whole-genome analysis. These three datasets offer researchers a unique opportunity to delve into the molecular-level pathological mechanisms of acute ischemic stroke and search for potential diagnostic biomarkers.

### 2.2 DEGs and enrichment investigation

The Limma R package was utilized to compare and analyze differences between 59 IS subjects and 44 healthy controls from datasets GSE16561 and GSE22255. Limma employs traditional Bayesian data analysis to filter frequencies. The significance level was set at an adjusted *p*-value of <0.05 and a log Fold Change (logFC) greater than 1. A DEGs heatmap can be generated using appropriate heatmap software. We conducted enrichment analysis of Gene Ontology (GO) functions using the R package clusterspectrum, and Kyoto Encyclopedia of Genes and Genomes (KEGG) enrichment analysis of associated genes. Through Metascape cluster analysis,^2^ we identified three significantly enriched GO keywords (*p* < 0.05) and pathways (*p* < 0.05).

### 2.3 Construction of PPI network

A PPI network was constructed using differential genes selected from the String database.^3^ The minimum interaction score required for the PPI network was set to 0.6. Additionally, individual outliers were excluded during the construction of the PPI network.

### 2.4 Random forest DEGs screening

In the study, we employed a random forest model to identify DEGs associated with IS. Regarding model parameterization, we utilized the Random Forest software for model creation, adjusting parameters such as the number of nodes and trees to optimize model performance. During the training process, we conducted gene feature selection, choosing the most predictive gene subset, and ensured model accuracy in distinguishing IS patients from the control group through model evaluation. This systematic approach not only optimized the structure of the random forest model but also enhanced the accurate identification of IS-related DEGs, providing a reliable analytical foundation for unraveling the pathogenic mechanisms of IS.

DEGs Random Forest models were created using Random Forest software. First, we estimated the average model error rate for all genes using out-of-range data. The ideal variable value for a binary node tree is 6, and for a random forest, it is 500. We then used the loss-of-precision method (the Gini method) to determine the effect size in the random selection forest pattern. Genetic disease factors with a significance score greater than 2.0 were selected for subsequent model development. The merged dataset’s unstructured hierarchical clustering of 10 key genes was categorized, and a heatmap was created using the freely accessible pheatmap program.

### 2.5 Artificial neural network modeling

When conducting neural network training, we integrated the GSE16561 and GSE22255 datasets and standardized the data using the R package ‘neuralnet’, ensuring consistent input ranges across all features. Subsequently, we constructed a feedforward artificial neural network for IS classification. Through iterative experiments and adjustments, we determined a network architecture that balanced complexity and performance: one hidden layer containing five neurons. The model architecture consisted of an input layer, a hidden layer, and an output layer. The input layer had neurons corresponding to the number of gene features in our dataset. The hidden layer comprised five neurons, and the output layer contained two neurons representing the binary classification outcomes—control (con) and treatment (treat). We employed the sigmoid activation function (logistic function) for both the hidden and output layers due to its suitability for binary classification tasks. The neural network was trained using the resilient backpropagation with weight backtracking (Rprop+) optimizer, which adjusts weight updates based on the sign of the gradient, improving convergence speed and stability. The sum of squared errors (SSE) was used as the loss function to measure the discrepancy between predicted outputs and actual targets. Training continued until convergence criteria were met, with a default maximum of 100,000 epochs, although training often stopped earlier when the partial derivatives of the error function fell below 0.01. We initialized the weights randomly within the range [−1, 1] and set a random seed of 12,345,678 to ensure reproducibility. To assess the model’s performance, we computed the validation results using the area under the receiver operating characteristic curve (AUC) with the ‘pROC’ software package. This metric comprehensively considers the model’s true positive rate and false positive rate, ensuring superior performance and generalizability. The disease class scores were deduced by utilizing the network’s predicted probabilities. The objective of this systematic approach was to optimize the structure and parameters of the neural network, ensuring the model’s effectiveness and robustness in diagnostic tasks.

### 2.6 AUC evaluation

The effectiveness of the scoring model on healthy controls and IS samples was evaluated using the GSE58294 dataset. ROC curves were constructed and the area under the curve was calculated using the proc. software package to measure classification efficiency. Additionally, a threshold for the ROC curve was determined, along with the specificity and sensitivity for identifying IS and healthy controls below this threshold.

### 2.7 Immune landscape estimate and correlation test

Using the R program ‘complot’ and 1,000 permutations, we derived 22 samples from the IS cohort using CIBERSORT^4^. We compared the transcript ratios of core leukocyte marker matrix genes (LM22) to analyze the value of immune cells. We selected cases with a CIBERSORT score of *p* < 0.05 or above for further study. To demonstrate variations in immune cell infiltration between the two groups, we created violin plots in R using the ‘vioplot’ package. We explored the relationship between the discovered gene indication and the number of invading immune cells through Spearman’s correlation study in R. Finally, we visualized the obtained correlations using the ‘ggplot2’ package’s charm technique.

## 3 Results

### 3.1 DEGs identification

**Figure 2:**
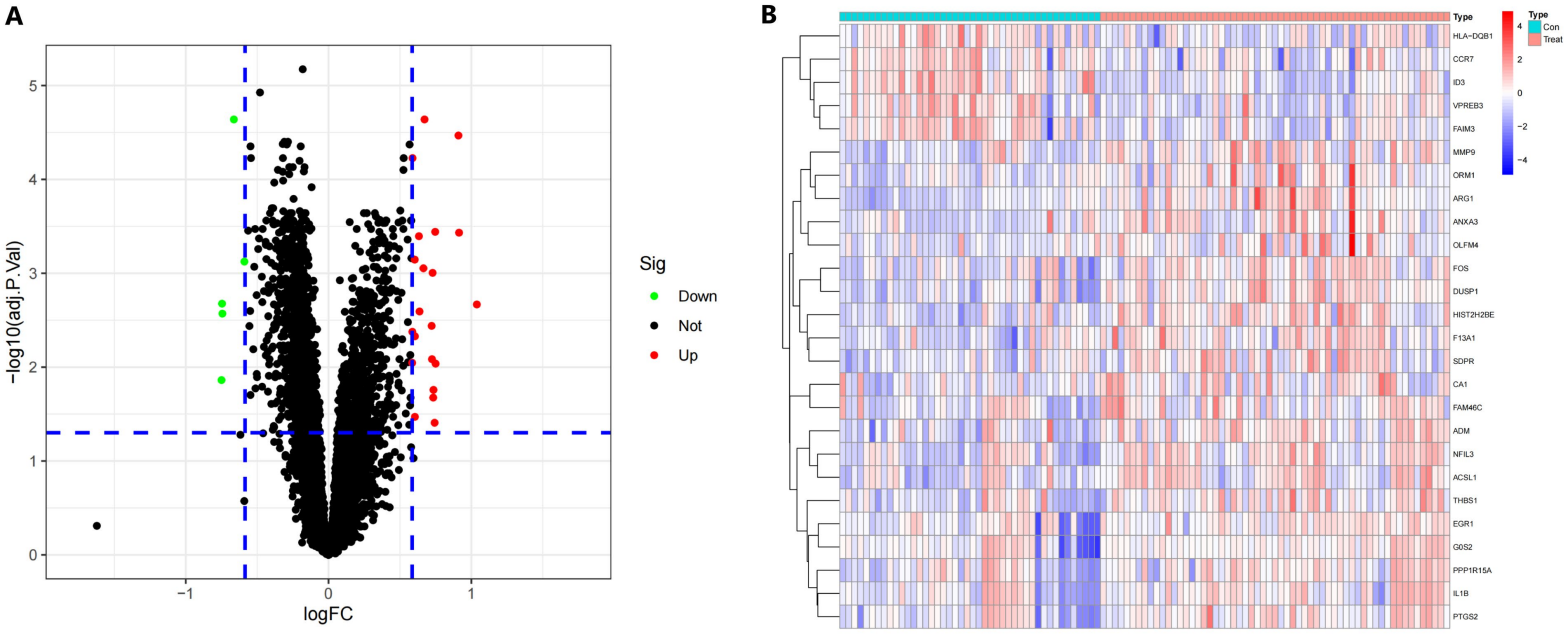
**(A)** A volcano plot displaying the findings of differential expression. Black dots represent the remaining functioning genes. **(B)** A heatmap in degrees. The colors on the chart vary from red to green, indicating strong to low expressiveness. The red bars in the heatmap’s top half reflect sick samples, whereas the blue bars represent healthy samples.

Limma’s technique was used to identify DEGs between samples from the IS dataset and healthy control samples. Bayesian testing was employed for this purpose. The DEGs data were visualized in a volcanic map (Figure 2A) and a heatmap (Figure 2B). Using a significance criterion of *p* < 0.05 and fold change values greater than one, the analysis identified 26 significant DEGs associated with IS (Supplementary Table S1).

### 3.2 DEGs metascape analysis

**Figure 3:**
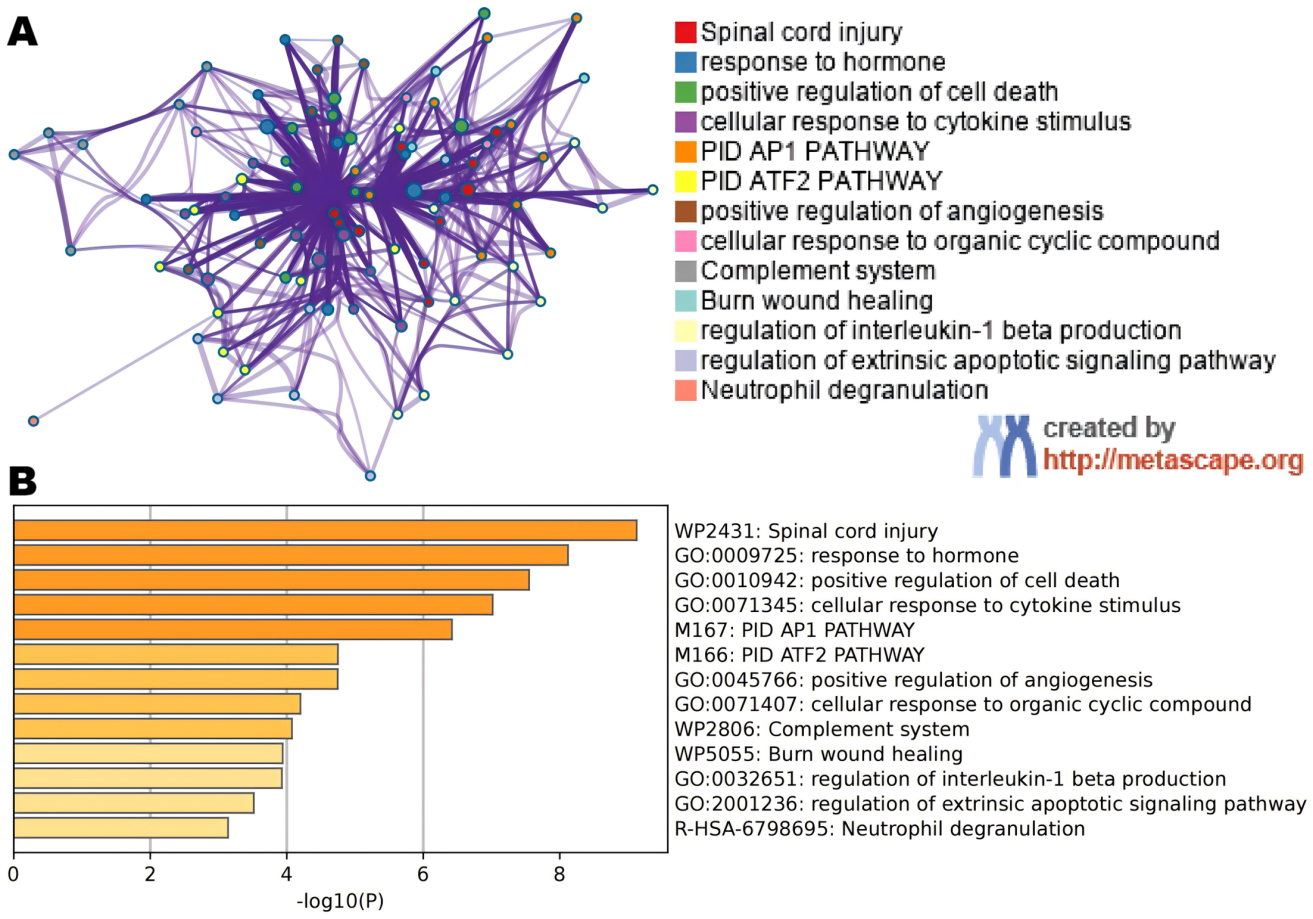
**(A)** An improved term network. Notes are colored using cluster IDs, and notes with the same cluster ID are frequently near to one other. **(B)** Colored bar plot of *p*-value for enlarged DEGs phrases.

To enrich and assess different genes, we utilized the Metascape database. We employed various resources such as GO Biological Processes, KEGG Pathways, Canonical Pathways, Cell Type Signatures, Responder Gene Sets, CORUM, TRUST, DisGeNET, PaGenBase, Transcription Factor Targets, WikiPathways, PANTHER Pathways, and COVID to enhance the lists of DEGs. Our enrichment background included every gene in the genome. We combined entries that had a *p*-value <0.01, a seed size of 3, and a contribution factor greater than 1.5. These entries were then ranked based on their membership commonality. Figures 3A,B display the top 13 terms from the Matescape enrichment study. Additionally, the findings of the route and process enrichment investigations can be found in Supplementary Table S2.

### 3.3 Enrichment analysis in IS patients’ and healthy control people’s samples

**Figure 4:**
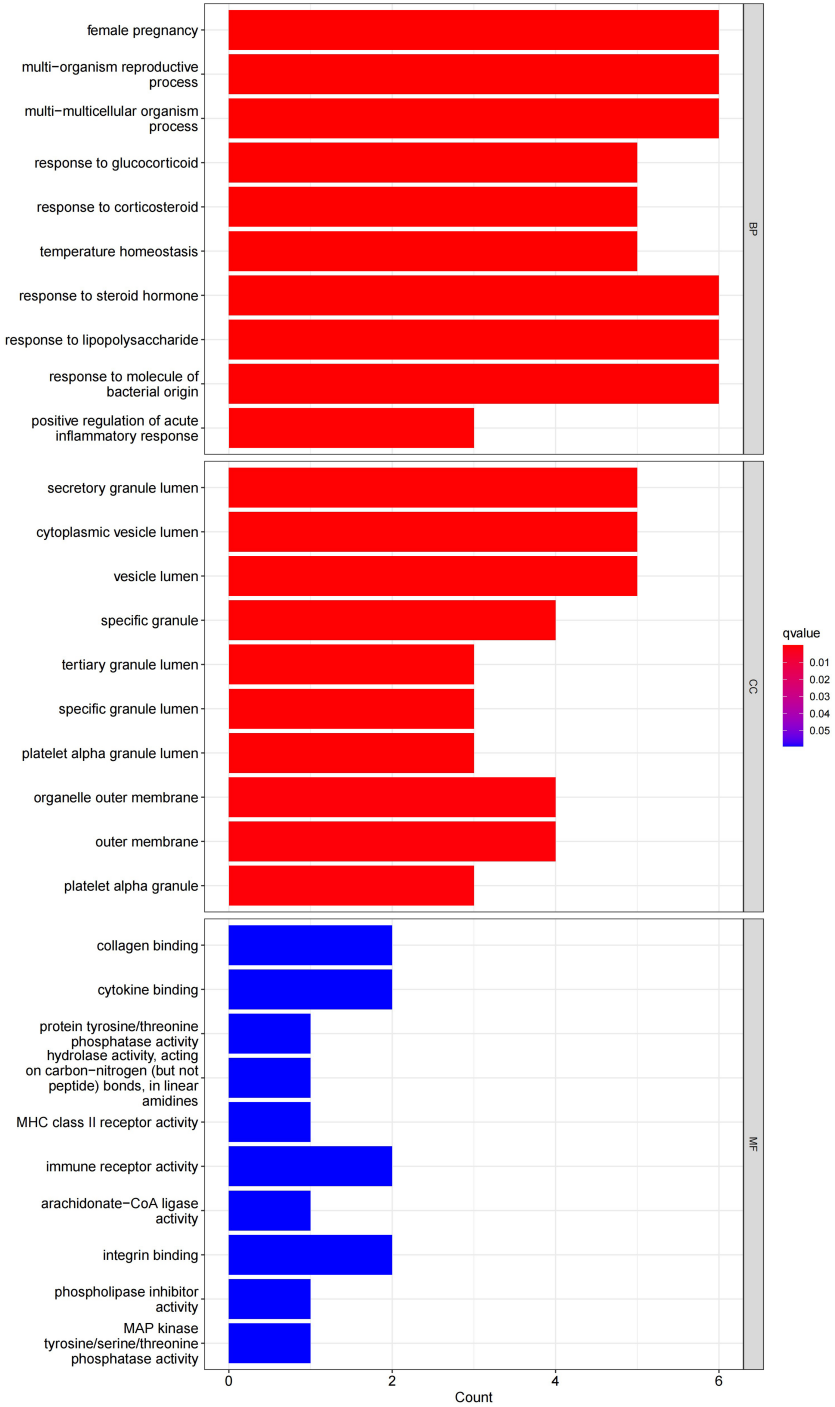
Graph displaying the results of the enrichment analysis. A bar graph is produced as a result of GO enrichment. The log 10 (adj *p*) values are represented on the y-axis, while the z-scores are plotted on the x-axis.

**Figure 5:**
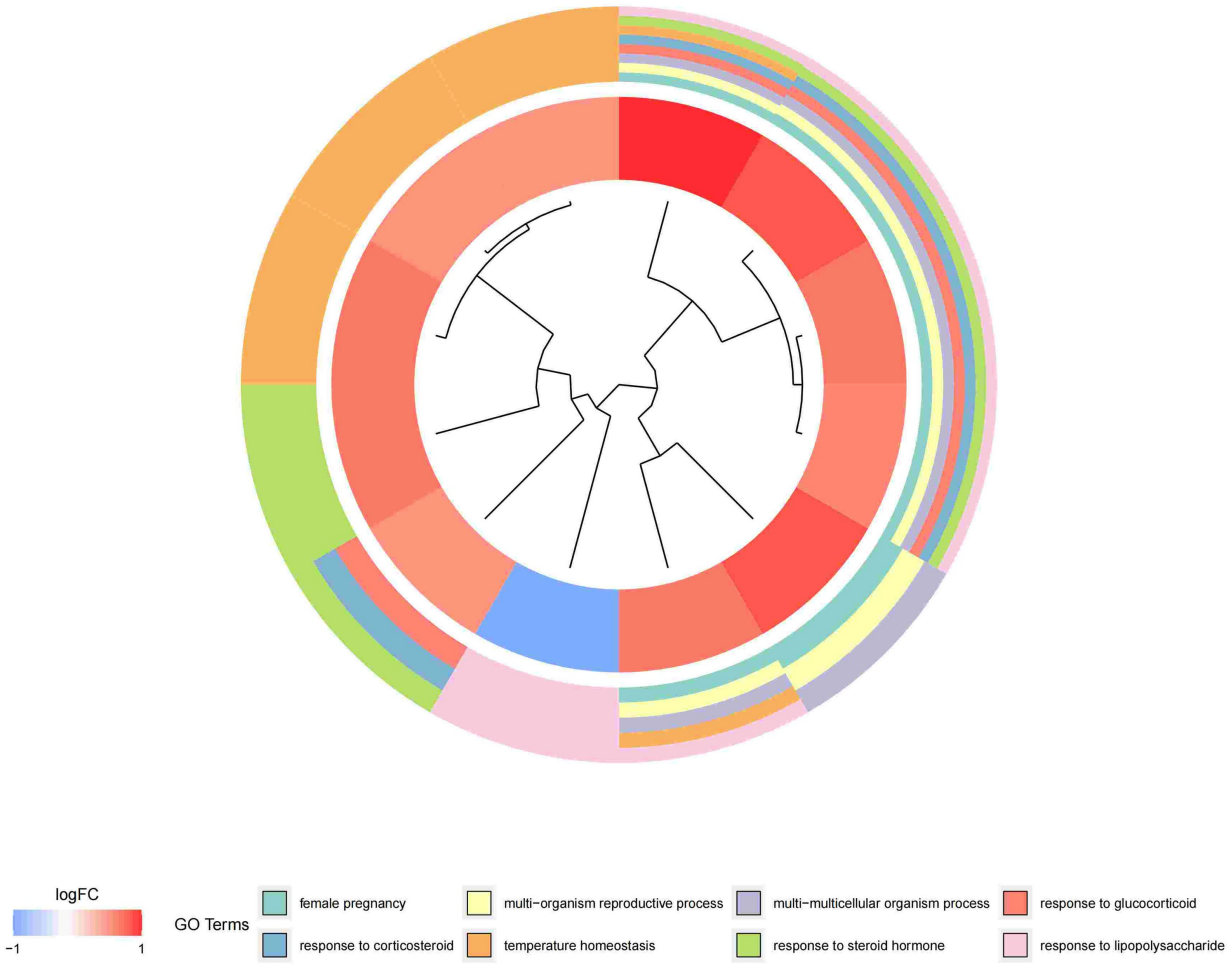
Graph displaying the results of the enrichment analysis. Gene clustering circles, with the inner circles representing DEGs, the red circles representing up-regulated genes, the blue circles representing down-regulated genes, and the outside circles representing GO keywords.

**Figure 6:**
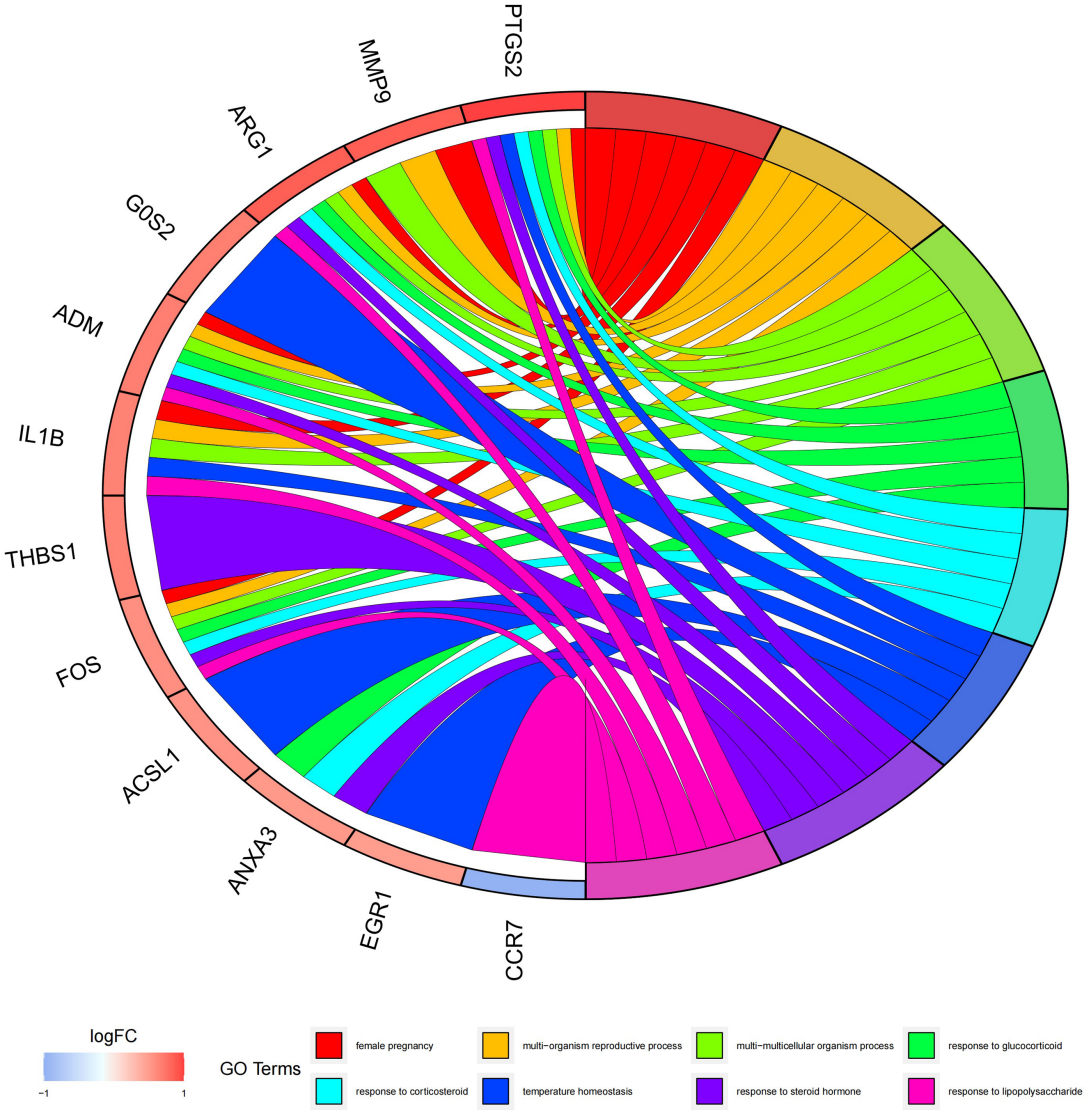
Graph displaying the results of the enrichment analysis. GO enrichment circle map. On the left are DEGs, with red bands indicating up-regulated genes and blue bands representing down-regulated genes. The various colored ribbons on the right indicate various GO ideas. Connecting lines represent genes that are included in GO terms.

**Figure 7:**
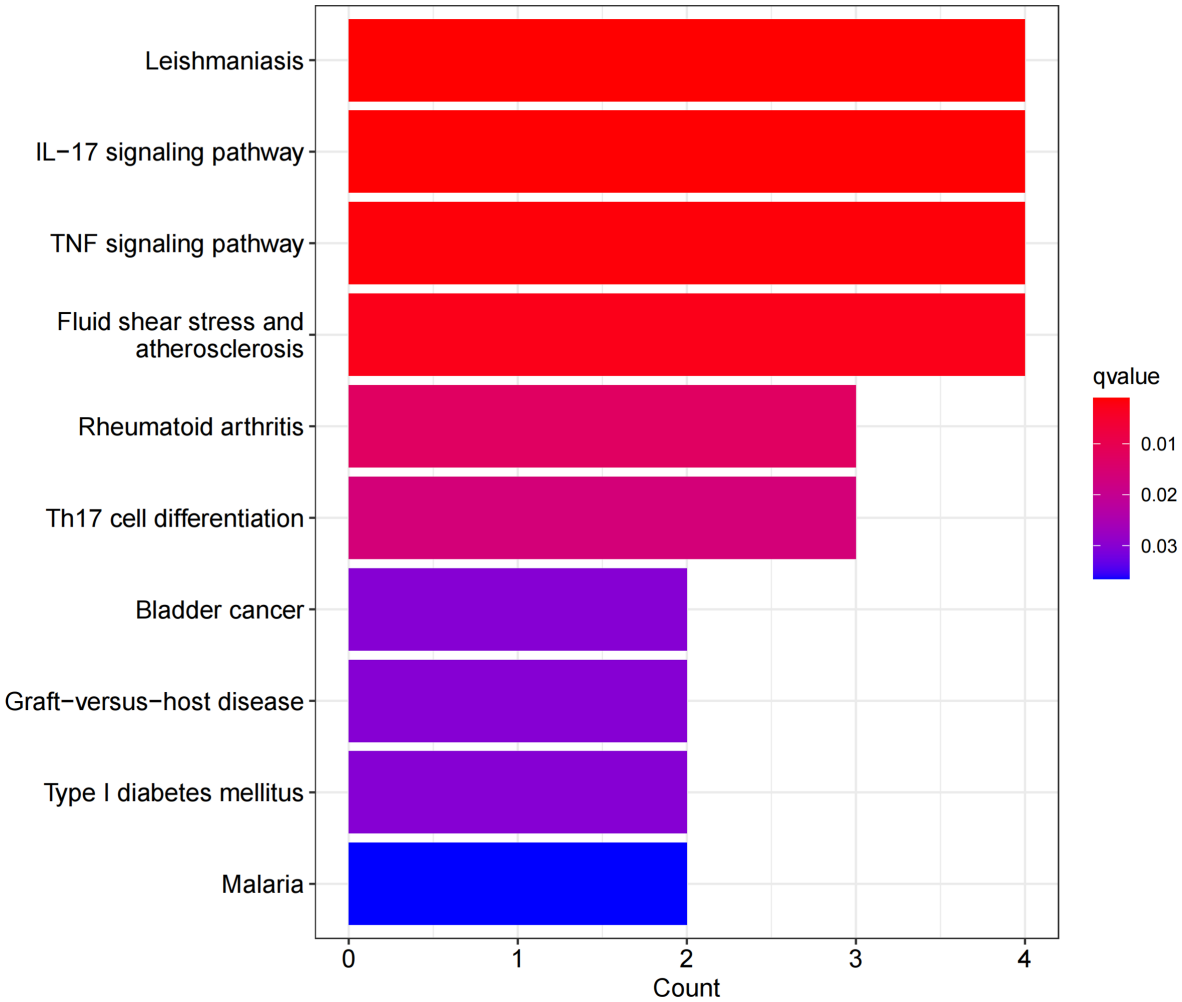
Graph displaying the results of the enrichment analysis. A bar graph displaying the findings of KEGG pathway enrichment. The log 10 (adj *p*) values are represented on the y-axis, while the z-scores are plotted on the x-axis. A bar graph represents the KEGG pathway, and the size of the histogram shows the number of genes in the route.

**Figure 8:**
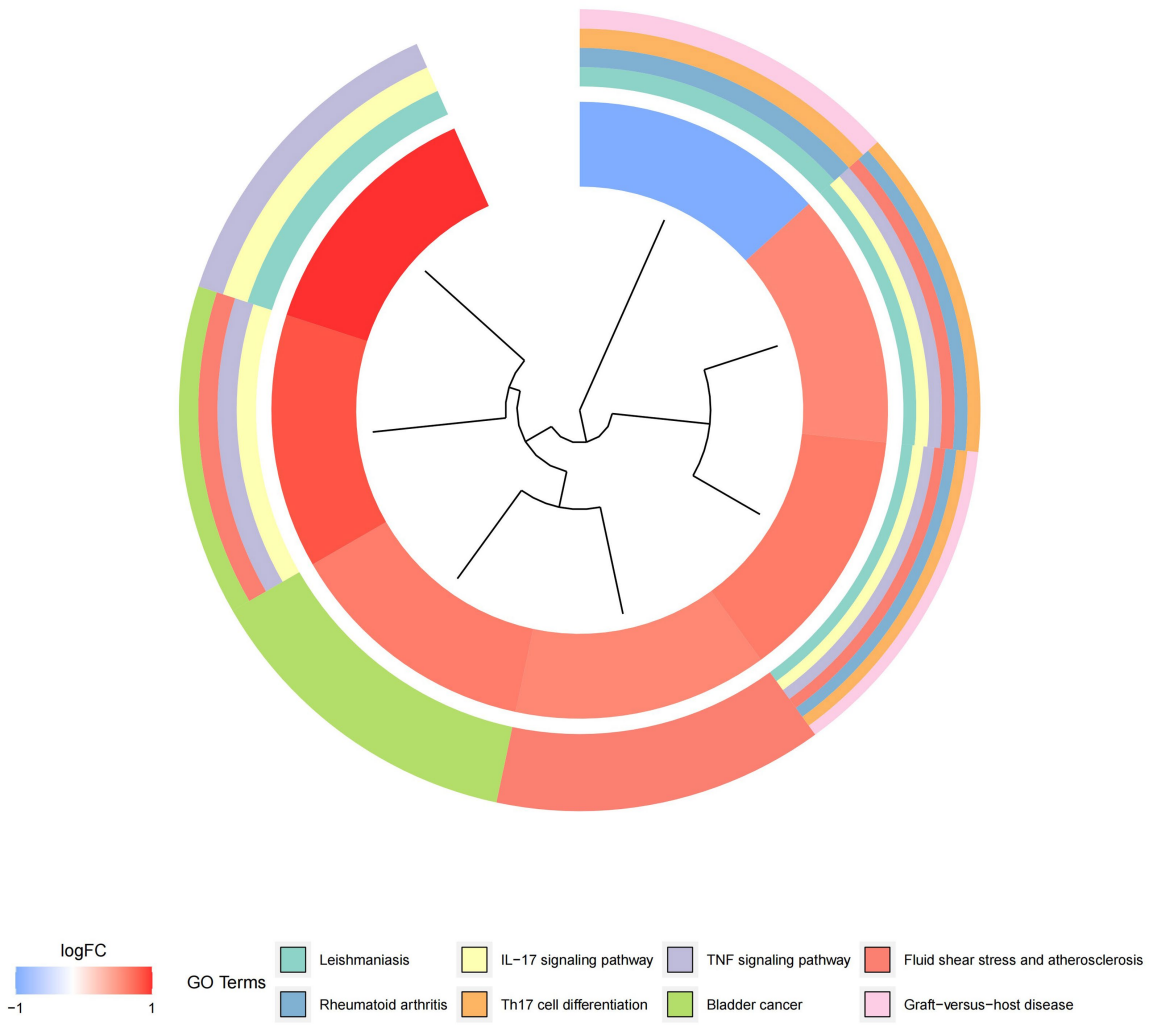
Graph displaying the results of the enrichment analysis. Gene clustering circles: the red circles represent up-regulated genes, the blue circles represent down-regulated genes, and the outside circles represent KEGG elements.

**Figure 9:**
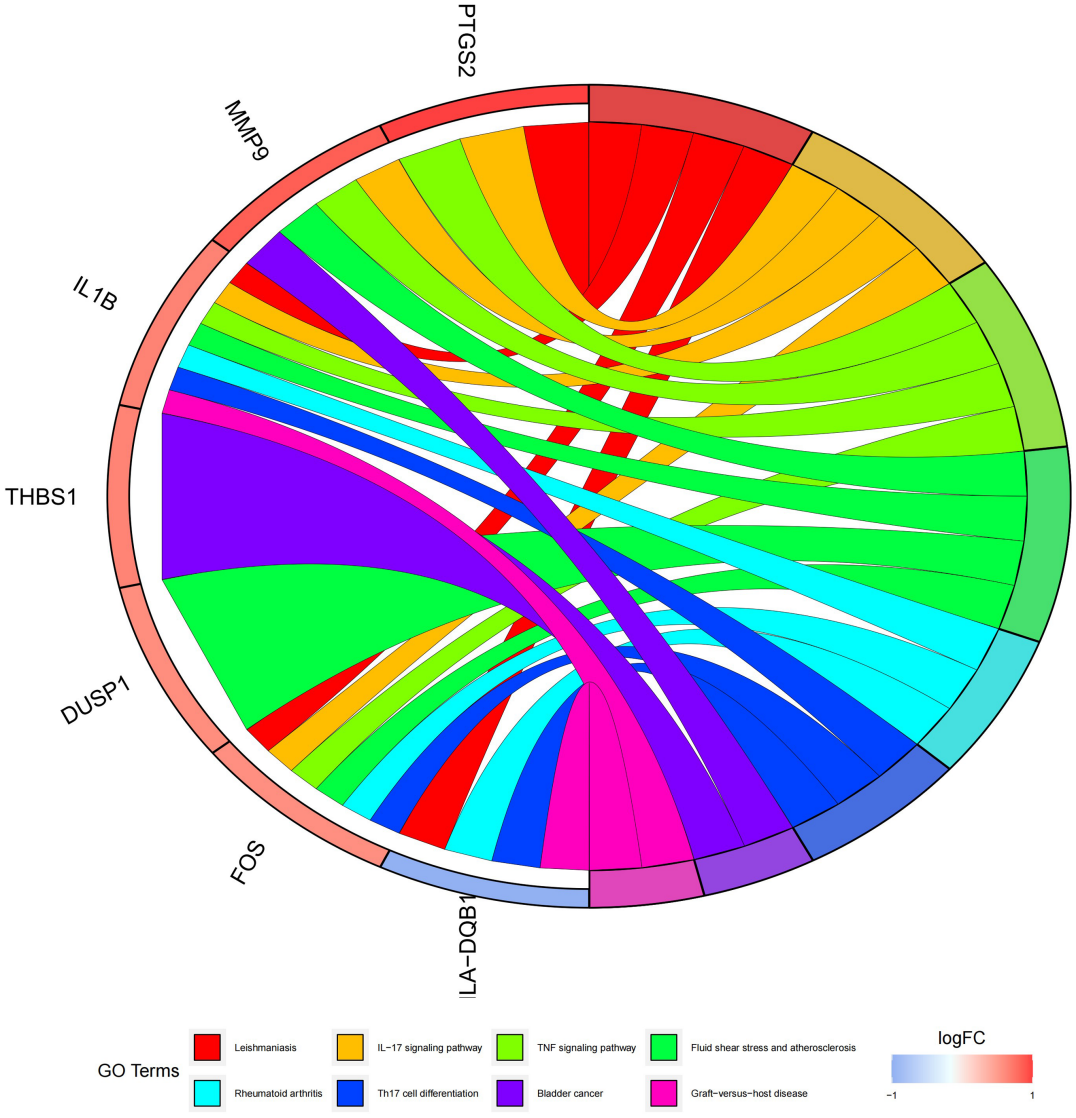
Graph displaying the results of the enrichment analysis. Diagram of KEGG pathway enrichment. DEGs are depicted on the left, with red bands representing up-regulated genes and blue bands representing down-regulated genes. On the right, different colored ribbons represent different pathways. Connecting lines reflect the roles of genes in this pathway.

In this study, we analyzed 26 significant DEGs using cluster analysis software to determine GO enrichment. The Benjamini-Hochberg correction was applied with P and Q values set at 0.05. To ensure unique GO-enrichment data, we compressed terms and removed phrases with >0.75 gene overlap. The results for the three GO enrichment zones are presented in Figures 4–6. Figure 4 showcases the GO enrichment findings for all three categories, highlighting -log10 (adj *p*) >5 GO words. The findings suggest that IS is associated with various linked biological processes such as multicellular organism process, reaction to steroid hormone, and response to lipopolysaccharide. Additionally, cellular components such as secretory granule lumen, cytoplasmic vesicle lumen, and vesicle lumen are involved. Molecular functions like immune receptor activation and other essential actions are also implicated. Figures 5, 6 provide further details on the GO enrichment terms and the main DEGs involved. We performed a KEGG pathway enrichment analysis on the DEGs, identifying several significantly enriched biological pathways, including the IL-17 signaling pathway, TNF signaling pathway, and fluid shear stress and atherosclerosis. Figures 7–9 present the results of these pathways, along with the associated DEGs.

### 3.4 Random forest tree selection

**Figure 10:**
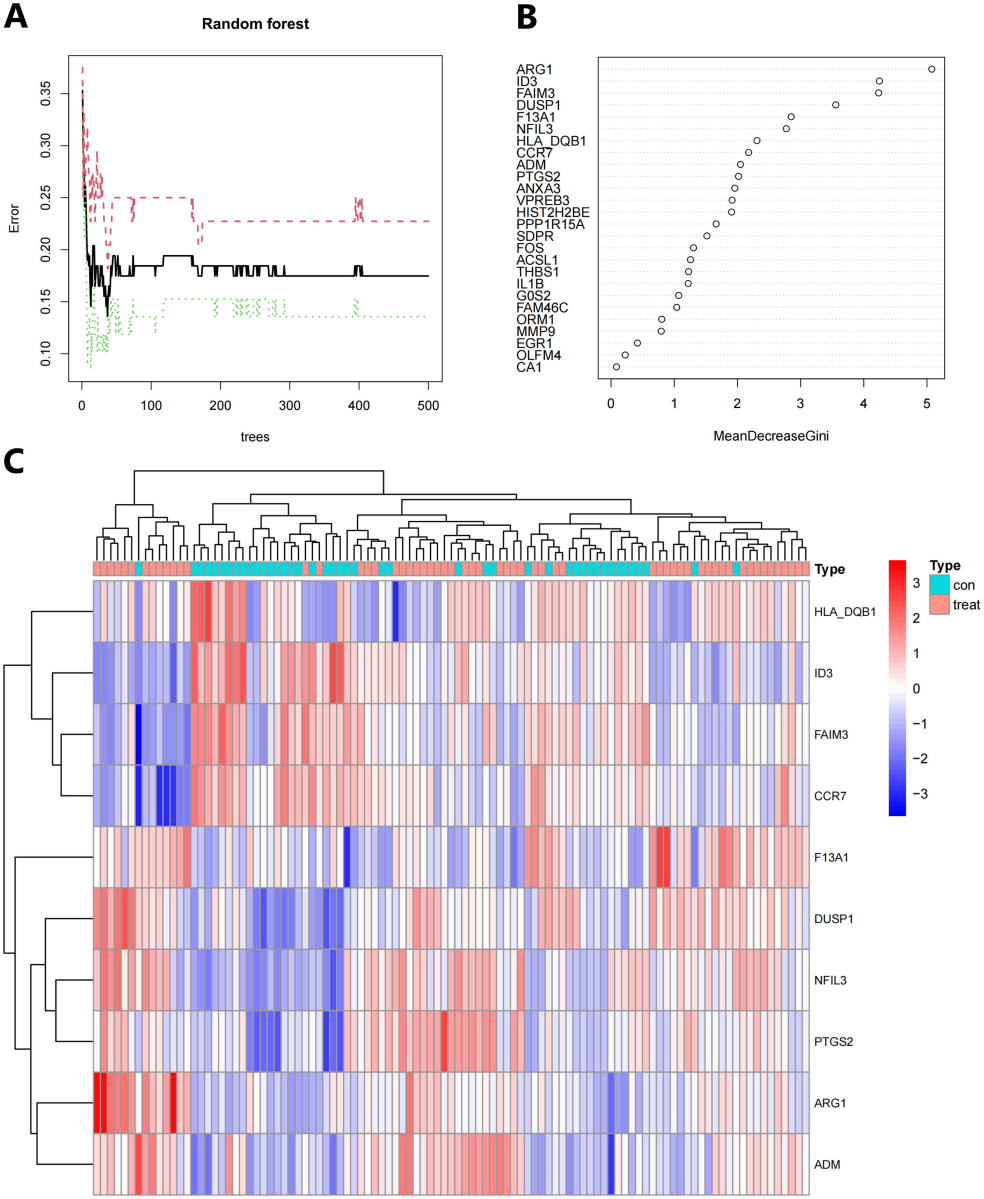
**(A)** The number of trees used influences the mistake rate. The x-axis represents the number of decision trees, while the y-axis represents the mistake rate. **(B)** To obtain the random forest classifier results, use the Gini coefficient approach. **(C)** Unsupervised clustering heatmap displaying hierarchical clustering of 10 important genes created by random forest when the GSE16561 and GSE22255 datasets were combined. Normal samples are represented by the red bands above the heatmap, while IS samples are represented by the blue bands. Red genes have high expression levels in the samples, whereas blue genes have low or undetectable expression levels in the samples.

The random forest method produces a score of 26. To determine the appropriate parameter mtry, we conducted recurrent random forest classification using all possible values from factors 1 to 26. We evaluated the model’s average error rate, which helps determine the optimal number of factors within a node to describe a binary tree. We selected 10 as the number of variables for the analysis. We minimized variants and ensured minimal out-of-band mistakes. For the final model, we used 500 trees as variables, based on the correlation plot of model uncertainty versus the number of selected trees (Figure 10A). Throughout the development process of the random forest model, we evaluated the variable correlation of the output scores using the Gini method. We assessed the accuracy and root mean square error, which are presented in Supplementary Table S3 as the main output results. From this evaluation, we selected 10 candidate genes with a significance larger than 2.0 for further investigation. In Figure 10B, we highlight DUSP1, ADM, FAIM3, ARG1, NFIL3, PTGS2, F13A1, HLA-DQB1, ID3, and CCR7 as the most important variables among the 10. With these 10 essential characteristics, we performed k-means unsupervised clustering on the pooled dataset. As shown in Figure 10C, these 10 genes can be used to distinguish between sick and normal samples. HLA-DQB1, ID3, FAIM3, and CCR7 are a group of genes that showed little or no positive control in the treated samples. On the other hand, F13A1, DUSP1, NFIL3, PTGS2, ARG1, and ADM belonged to different clusters and exhibited high expression in healthy samples and low expression in diseased samples, see Additional file 4 for the codes used.

### 3.5 Creating a model of an artificial neural network

**Figure 11:**
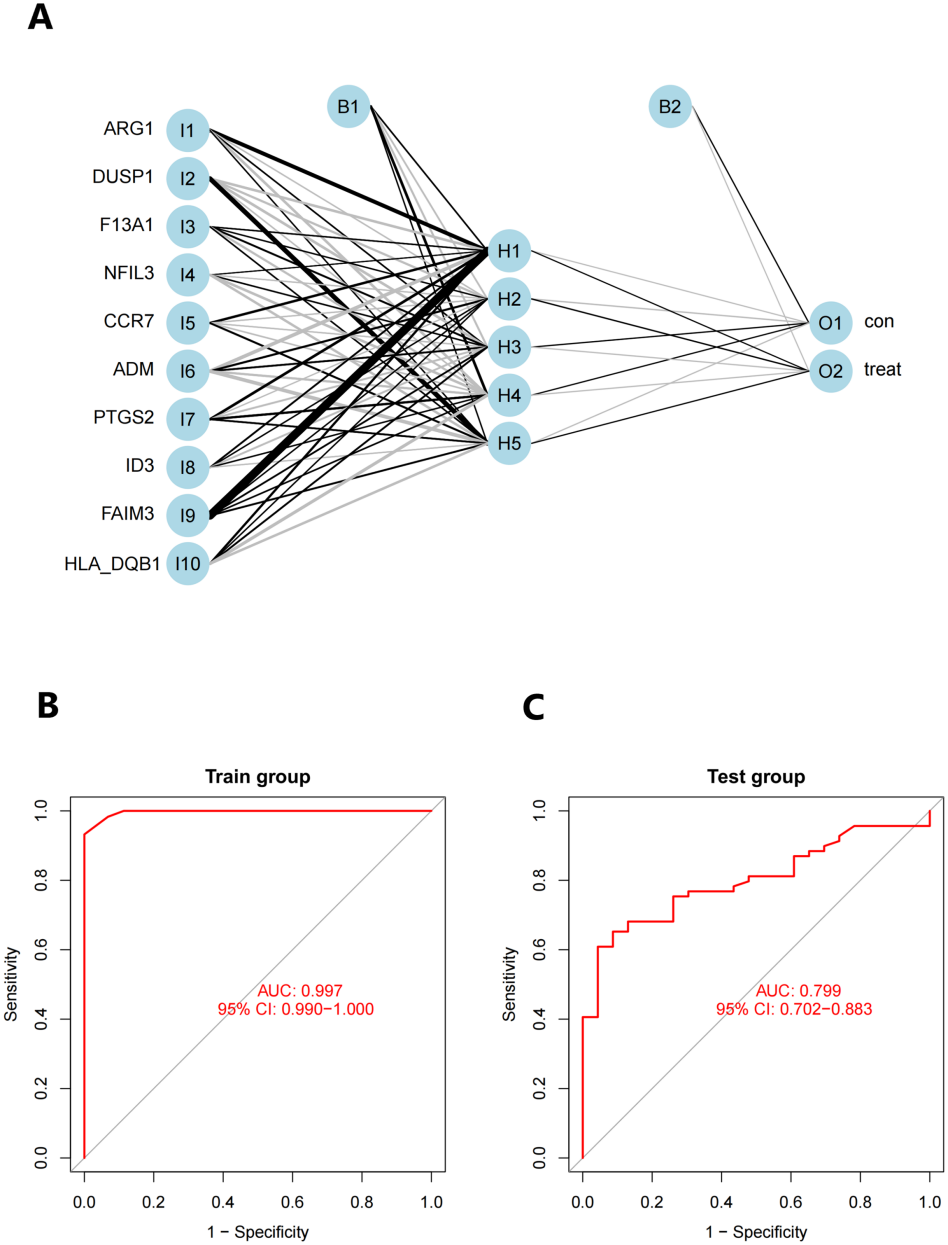
**(A)** Visualization of a neural network. **(B)** Training set for validating ROC curve results (merged dataset of GSE16561 and GSE22255). **(C)** The testing team examines the ROC curve results (combined dataset of GSE58294).

The neural network software utilizes the combined datasets of GSE16561 and GSE22255 to create artificial neural network models. The first step in data normalization is data preparation. The min-max approach [0, 1] is used to separate the amplified information prior to training the network. Before starting the computation, the maximum and minimum data values should be normalized, and the number of hidden layers should be set to 5. There are no strict guidelines for determining the number of layers and neurons to use as parameters. The number of neurons should be approximately two-thirds of the input layer and one-third of the output layer. Therefore, the number of neurons parameter is set to 10. The training group aims to determine the value of each candidate’s DEG. The validation set is used to evaluate the classification performance of the model in terms of gene expression and gene weights. The rank value of the resulting illness neural network model is calculated as the sum of Gene Expression multiplied by Neural Network Weight (Figure 11A). We utilized all of the available data to build the neural network model. The experimental findings indicate that the model’s area under the ROC curve (AUC) is close to one (average AUC > 0.99), suggesting its resilience. We conducted a review of the GSE58294 datasets to ensure that the area under the ROC curve (AUC) remained around 0.9 (Figures 11B,C), see Additional file 5 for the codes used.

### 3.6 The immune landscape and the features of IS patients

**Figure 12:**
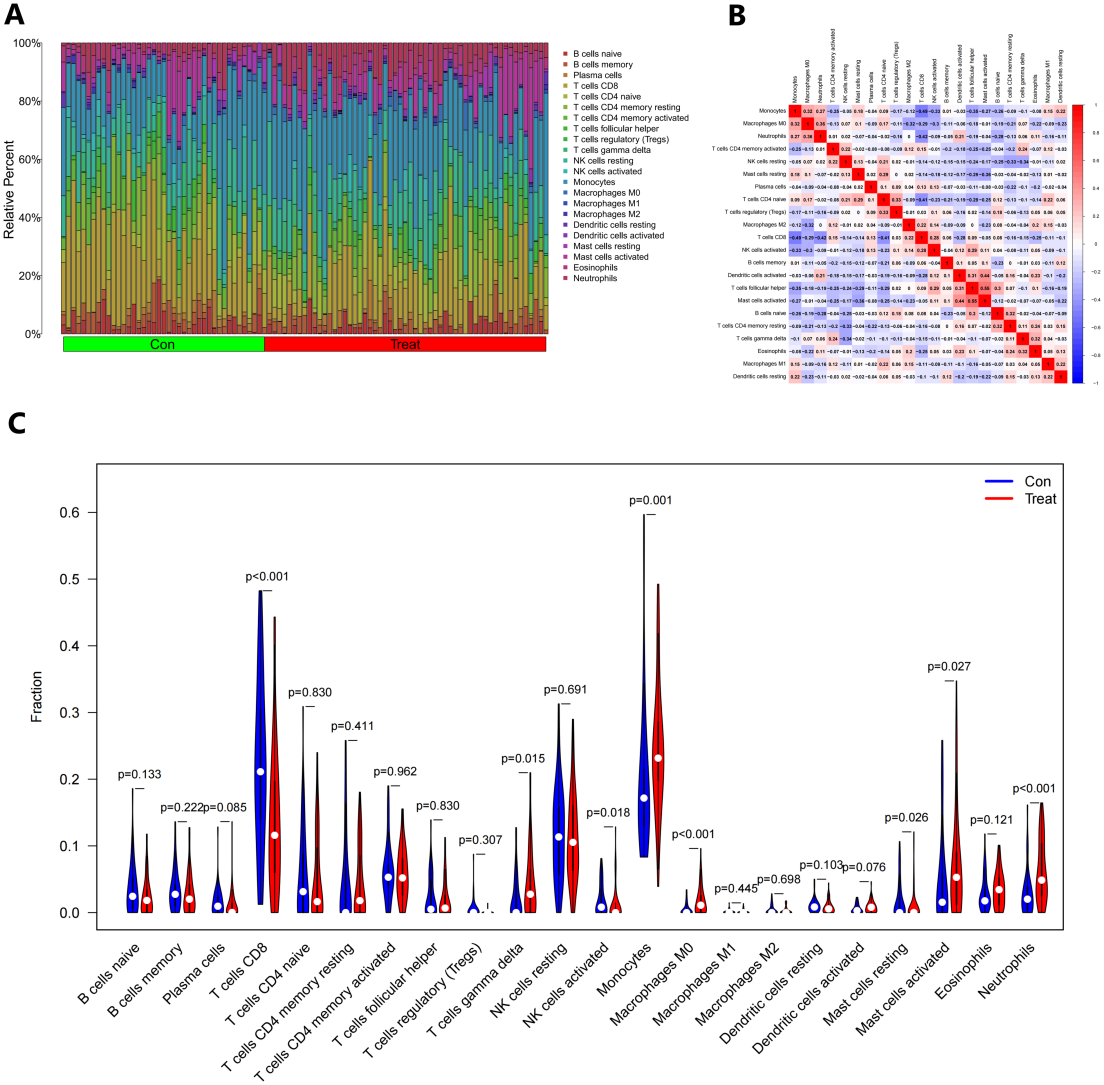
The immunological landscape of IS. **(A)** The CIBERSORT algorithm was used to forecast the proportions of 22 immune-cell types in the control and treatment groups. **(B)** Immune cell infiltrating correlation analysis. **(C)** Analysis of 22 immune-cell subsets in the control and treatment groups.

According to functional enhancement analyses, immune-related networks were found to be elevated in samples from individuals with IS compared to healthy controls. To investigate changes in the immunological state between IS patients and healthy controls, we analyzed genomic information from blood samples obtained from the GSE16561 and GSE22255 pooled datasets. The CIBERSORTx tool was utilized to calculate the percentages of 22 different types of immune cells in the data. CIBERSORTx is an online tool that uses a background subtraction technique to determine the relative abundance of immunological tissues in individuals. Figure 12A illustrates the distribution of these 22 unique immune cell types in participants with IS and healthy controls. By employing Spearman’s correlation analysis, we examined the associations between immune cells. The strongest positive association (*R* = 0.55) was observed between T cells follicular helper and activated Mast cells, while the most significant negative correlation (*R* = −0.49) was found between Monocytes and T cells CD8 (Figure 12B). Furthermore, the fraction of T cells CD8 in the IS group was significantly lower (*p* < 0.001) compared to the non-IS group (Figure 12C).

## 4 Discussion

Compared to previous bioinformatics studies on disease mechanisms, this study has several advantages. Firstly, it utilizes multiple data packages in the R language to rigorously normalize the data through background calibration, normalization, elimination of batch effects, and removal of outliers. This ensures the reliability of the results to the greatest extent possible. Secondly, the study employs Random Forest and Artificial Neural Network techniques to identify disease-related module genes for further investigation. Random Forest and Artificial Neural Network have the advantage of discovering highly linked genes and clustering them into gene modules. These resulting modules can then be correlated with clinical parameters for follow-up investigation. Additionally, this study takes a multi-bioinformatics approach by combining multiple biomolecules to analyze disease mechanisms. This comprehensive and in-depth analysis aims to explore the underlying mechanisms of IS in a more comprehensive and in-depth manner.

In this study, we first calculated the DEGs associated with IS using the classifier model and identified 10 key candidate DEGs. Then, we utilized the neural network model to calculate the anticipated weights of the chained genes, generate the neural IS classification model score, and evaluate the classification performance of the model using autonomous sample datasets. The AUC efficiency was found to be exceptional, indicating that neural IS has a high classification efficiency.

ARG1 is a cytoplasmic enzyme primarily expressed in the liver, but it is also found in immune cells in peripheral blood. It plays a crucial role in the urea cycle and is involved in the immune response following organismal injury. Additionally, ARG1 is closely associated with recovery from IS (10–12). Jickling’s study demonstrated consistent upregulation of ARG1 mRNA in leukocytes of IS patients. Signaling downstream of the injured brain upregulates ARG1 mRNA levels in immune cells in peripheral blood and downregulates the expression of miR-30a-5p, which is further enhanced by miR-30a-5p downregulation (13). In an animal model, Cai discovered that ARG1 promotes microglia/macrophage cytomegaly and inflammation regression in stroke mice, thus contributing to brain tissue injury repair (14). Zhu, through bioinformatics analysis, identified differences in the expression of ARG1 and Kruppel-like factor12 (KLF12) genes in IS and normal specimens, suggesting a potential association with the occurrence of IS (15). DUSP1 plays a crucial role in regulating inflammation and the immune response. It is involved in various cellular processes, including T cell differentiation, development, and activation. Additionally, DUSP1 has been associated with the development of several autoimmune diseases (16). A study by Li found that DUSP1 was overexpressed in both males and females, as well as in both elderly and young individuals with IS (17). Another study by Xu demonstrated that DUSP1 reduces ischemic reperfusion injury in the brain by inactivating the JNK-Mff pathway and inhibiting mitochondrial fission, thereby attenuating cerebral ischemia–reperfusion injury (18). Furthermore, DUSP1 has been suggested as a potential biomarker and therapeutic target for interfering with the inflammatory immune response of macrophages induced by ischemia-hypoxia (19). The F13A1 gene encodes the A chain of human coagulation factor XIII, which plays a crucial role in covalently cross-linking fibrin fibers and stabilizing fibrin clots (20). It is involved in various physiological processes, including coagulation, wound healing, angiogenesis, and platelet degranulation. Functional abnormalities or mutations in the F13A1 gene can lead to the development of multiple disorders (21). The presence of the F13A1 204Phe allele has been closely associated with IS in young women, and the risk of IS is further increased when combined with the use of oral contraceptives. Additionally, the presence of this allele may serve as a prognostic indicator for IS (22). NFIL3, a basic leucine zipper transcription factor, is expressed in multiple immune cells and plays a crucial role in regulating immune function. Studies conducted by Tamai have demonstrated that NFIL3 exhibits neuroprotective properties, promotes neuronal survival, and has anti-apoptotic effects. Additionally, NFIL3 is involved in various cellular processes including immune cell development, cell survival, and circadian rhythm control (23). CCR7 helps T lymphocytes to enter chemokine receptors in lymph nodes and plays an important role in the human immune system. It was found that CCR7 expression was significantly downregulated after ischemic brain white matter injury, thereby reducing homing migration of DCs (dendritic cells) and inhibiting antigen-dependent T lymphocyte expansion, which in turn failed to respond to antigen-specific immune responses, suggesting that CCR7 may play an important role in IS (24, 25). Yang found that CCR7 expression was significantly up regulated in the serum of IS patients, which may have an important impact on the changes in the disease (26). In addition, CCR7 expression was found to be upregulated in astrocytes and granule layer neurons in the CA1 region of the hippocampus in a gerbil model of transient localized cerebral ischemia, and the timing of CCR7 expression in both cells correlated with the course of the disease (27). ADM is a cytokine closely linked to vascular function. It is produced by various tissue cells, such as endothelial and vascular smooth muscle cells. Because of its small size, ADM can easily move between the blood and the interstitium (28, 29). ADM has been shown to have several beneficial effects, including reducing peripheral blood pressure, preventing atherosclerosis, and maintaining endothelial cell stability. Hypertension, atherosclerosis, and vascular calcification are known to be high risk factors (30, 31). Hirose found a positive association between the rs3840963 polymorphic locus of ADM2 and the development of asymptomatic cerebral infarction and cerebral white matter lesions (32). PTGS2, also known as COX-2, plays a crucial role as an inflammatory mediator throughout the process of inflammation formation. Overexpression of PTGS2 can disrupt the internal environment balance, contribute to the inflammatory response after brain injury, and promote the expansion of the brain infarct area (33). Studies have shown that PTGS2 can mediate both early damage and late repair effects on neurons. Therefore, targeting PTGS2 could be a potential therapeutic approach to alleviate neurological damage caused by cerebral ischemia (34, 35). ID3 is associated with vascular disease pathology and plays a crucial role in various cellular processes. It also has a protective effect against atherosclerosis, and polymorphisms in the ID3 gene are considered potential risk markers for human atherosclerosis (36). Zhang et al. found that ID3 inhibits bHLH protein-DAN binding and gene expression in B cells. Using bioinformatics analysis, investigators established a regulatory relationship between ID3 and IS (37). O’Connell et al. conducted a genome-wide expression profiling study using microarray analysis of peripheral blood from 39 patients with acute IS and observed significant expression of ID3 in these patients (38). FAIM3 is predominantly expressed in the digestive and urinary tracts, bone marrow, and testicular tissues, and is involved in homeostasis and activation of the innate immune system. However, it appears to be limited to the cerebellum in the nervous system (39, 40). Brennery et al. discovered that FAIM3 is essential for dendritic cell pro-inflammatory function and suppression of T-regulatory cell activation (41). HLA-DQB1, a paralog of the HLA class II *β*-chain, plays a crucial role in the immune system’s ability to differentiate between proteins produced by the body and those produced by external invaders like viruses and bacteria (42). Variations in HLA-DQB1 have been linked to muscular weakness, poor coordination, numbness, and various other health issues. Additionally, these variations increase the risk of inflammatory demyelinating illnesses in the central nervous system, specifically affecting the white matter of the brain (43). The exact mechanism through which the HLA-DQB1 gene influences the susceptibility to these illnesses is still unclear. However, it is important to note that other changes in both HLA and non-HLA genes, some of which remain unidentified, may also contribute to the development of complex disorders.

According to current research, the immune system plays a role in the progression of individuals with IS from the acute to the chronic phase (44). In Jayaraj’s study, it was found that various inflammatory cells, such as neutrophils, B cells, and monocytes, enter the ischemic zone after IS, leading to brain damage (45). The CD^+^_3_CD^−^_4_CD^−^_8_ T cells contribute to brain damage through the apoptosis-related factor ligand/protein tyrosine phosphatase non-type 2 receptor/tumor necrosis factor alpha pathway, which exacerbates neuroinflammation and brain injury (46). In the pathogenesis of IS, neuronal stromal cells and macrophages have a dual function. They promote the production of inflammatory factors, disrupt the blood–brain barrier, allow leukocytes to enter damaged brain tissue, and further exacerbate brain injury. Macrophages with different gene expression profiles have been found to have neuroprotective effects in different inflammatory settings (47). Our study suggests that T cells CD8, Macrophages M0, Neutrophils, T cells gamma delta, NK cells activated, Mast cells resting, and Mast cells activated may be related to the development of IS. Excessive infiltration of ischemic brain tissue by Neutrophils in patients with IS can lead to a systemic inflammatory response and disruption of the blood–brain barrier. Additionally, peri-infarct tissue neovascularization is equally important to the structure, along with reperfusion (48). Massive infiltration of T cells is a prominent characteristic of IS, where T cells directly interact with neurons and produce a significant amount of cytotoxic factors (49). However, further studies have revealed that transient immunosuppression also occurs after IS, and over time, CD^+^_8_ T lymphocytes may participate in the repair process following IS (50). Additionally, dendritic cells play a role in the immunological response to IS by enhancing antigen presentation by T cells and mast cells (51, 52). However, more experimental evidence is required to understand the specific mechanisms underlying these associations. These immune cells play a crucial role in IS and should be the focus of future research.

Using GEO datasets and the CIBERSORTx tool, we identified significant alterations in the immune microenvironment of IS patients, revealing complex interactions between these changes and key DEGs. A major immune feature observed was the substantial reduction in CD^+^_8_ T cells, which play a crucial role in adaptive immune responses. Their reduction in IS patients suggests a post-stroke immunosuppressive state, potentially impairing inflammation control and tissue repair. DEG analysis revealed that genes such as DUSP1 and ARG1 were significantly associated with the decrease in CD^+^_8_ T cells, indicating a role in modulating T cell function during IS. DUSP1, a negative regulator of inflammatory signaling pathways, may act by modulating T cell activity and reducing immune-mediated neuronal damage. In contrast, a marked increase in neutrophil infiltration was observed, indicating a strong inflammatory response during the acute phase of IS. Excessive neutrophil infiltration is known to exacerbate blood–brain barrier disruption and neuronal damage by releasing oxidative stress molecules and inflammatory mediators. ARG1, which was highly expressed in IS patients, is closely associated with macrophage polarization and may influence neutrophil recruitment and activation, further contributing to the inflammatory response seen in stroke. The downregulation of CCR7 in IS patients may impair the migration and antigen presentation of dendritic cells and T cells. As a key regulator of immune cell migration and antigen presentation, the downregulation of CCR7 may hinder immune cell recruitment and effective antigen presentation, affecting T cell proliferation and activation. This suggests that local immune responses, particularly in regions of white matter damage, may be suppressed, with CCR7 downregulation exacerbating neuroinflammation and pathological damage. Furthermore, the upregulation of ADM was linked to vascular function and inflammation regulation. ADM, a vasoactive peptide, plays a dual role in maintaining endothelial stability and modulating immune cell activity. Its increased expression was associated with enhanced macrophage and T cell infiltration, suggesting that ADM may help suppress excessive inflammation while promoting tissue repair in IS. In conclusion, this study highlights the intricate relationship between key DEGs and immune cell infiltration in IS. These findings suggest that DEGs may regulate immune responses and play a critical role in the onset and progression of IS. By integrating DEG analysis with immune cell profiles, we gained deeper insights into IS immunopathology, identifying potential targets for immune-modulating therapies. Future studies should focus on further validating the causal relationships between these genes and immune cell function and exploring their potential for personalized IS treatment.

In this study, we conducted an in-depth analysis of 26 significant DEGs using GO and KEGG enrichment analysis, revealing key biological processes, cellular components, and molecular functions associated with IS. GO enrichment analysis showed that IS is closely related to biological processes such as multicellular organism processes, steroid hormone response, and lipopolysaccharide response. The enrichment of these biological processes suggests that IS pathology involves widespread systemic responses, particularly in inflammation and immune regulation. For example, the link between steroid hormone-regulated processes and inflammation may reflect abnormal immune and inflammatory responses in IS patients. The enrichment of the lipopolysaccharide response further supports the idea that a strong immune response accompanies IS, indicating that infection and immune response may play a key role in the onset and progression of stroke. Additionally, the enriched cellular components, including secretory granule lumen, cytoplasmic vesicle lumen, and vesicle lumen, suggest potential roles in regulating intracellular and extracellular transport and intercellular signaling, which may be closely related to immune cell activation and function regulation. On the molecular function level, GO analysis revealed associations between IS and essential molecular functions such as immune receptor activation. Immune receptor activation is a critical mechanism in regulating immune responses and inflammation, suggesting that immune receptors play a crucial role in IS immune regulation, possibly by modulating immune cell activation and signaling pathways during stroke onset. Meanwhile, KEGG pathway enrichment analysis identified several significant biological pathways, including the IL-17 signaling pathway, TNF signaling pathway, and fluid shear stress and atherosclerosis pathways. The enrichment of these pathways reflects the critical role of pro-inflammatory signaling in IS, particularly the IL-17 and TNF signaling pathways, which are widely recognized as major drivers of inflammation. Activation of these pathways may contribute to neuronal damage and blood–brain barrier disruption by promoting neutrophil and other immune cell recruitment. Additionally, the enrichment of the fluid shear stress and atherosclerosis pathways suggests a strong connection between stroke and atherosclerosis, with endothelial dysfunction potentially playing a key role in IS pathogenesis. These enrichment analysis results highlight the importance of IS-related DEGs in regulating immune responses and suggest that these genes play a crucial role in the inflammatory response and tissue damage following stroke. These findings are highly relevant to our study’s focus, deepening our understanding of the molecular mechanisms underlying IS and providing potential therapeutic targets for future interventions. For example, the activation of the IL-17 and TNF signaling pathways points to the possibility of targeting these pathways to mitigate stroke-related inflammation and reduce neuronal damage. Additionally, the enrichment of immune receptor and vesicle-related cellular components suggests potential intervention pathways by regulating intracellular transport and signaling. Future research should further explore these genes and their associated pathways to validate their functional roles in stroke onset and progression, providing new directions for the development of personalized stroke treatments.

In our study, we conducted an in-depth exploration of the pathogenic mechanisms of IS by integrating the whole-genome expression profiles from three datasets (GSE16561, GSE22255, and GSE58294) and performing immune cell analysis. Initially, using the Limma package, we conducted differential expression analysis to identify the DEGs between IS patients and the control group. Subsequently, employing immune cell quantification tools such as CIBERSORT, we quantified the immune cell types, revealing relative abundance information across different samples. Through immune cell analysis, we identified significant immune cell changes between IS patients and the control group, encompassing various subgroups such as T cells, B cells, monocytes, and macrophages.

Further analysis unveiled the DEGs associated with immune cells in the context of IS, providing crucial insights into the immunological mechanisms underlying IS. Our study not only offers a profound understanding of the immunology of IS but also provides robust support for the development of future immunotherapeutic strategies targeting IS.

In this study, we utilized Random Forest and Artificial Neural Network models due to their robustness and ability to handle complex biological data. RF is particularly effective in gene selection, as it resists overfitting, while ANN captures non-linear relationships and optimizes classification through weight adjustment. To further evaluate the robustness of these methods, we compared them with other commonly used machine learning models such as Support Vector Machine and Logistic Regression. Our results demonstrated that the Random Forest and Artificial Neural Network models outperformed these traditional models, particularly in terms of prediction accuracy and area under the curve, highlighting the efficiency of our approach. Additionally, we performed cross-validation across multiple independent datasets from the GEO database (GSE16561, GSE22255, and GSE58294), which originated from various research institutions and represented different populations. Despite these differences in sample characteristics, Random Forest and Artificial Neural Network models consistently achieved high accuracy across all datasets, further confirming the generalizability of our method. Thus, by comparing Random Forest and Artificial Neural Network models to other machine learning techniques and validating their performance on external datasets, we ensured the robustness and reliability of our results, enhancing the overall credibility of the study.

This study addresses several key issues. First, by comparing tissue samples from IS patients and healthy controls, we identified DEGs, providing valuable insights into the molecular mechanisms of IS. However, a challenge in clinical application is the time required for RNA extraction and quantification of DEGs, which may not align with the treatment window for acute IS. This could potentially delay treatment decisions. Thus, while identifying DEGs is crucial for understanding disease mechanisms and discovering potential biomarkers, its practical use in rapid diagnosis remains constrained by time limitations. Moreover, this study employed a combination of Random Forest and ANN models for IS diagnosis prediction. Although initial results demonstrate high predictive accuracy, further validation is needed to ensure clinical applicability across diverse patient populations. Future research should focus on optimizing these diagnostic models and exploring more efficient analytical methods to ensure their reliability and practical utility in clinical settings.

## 5 Conclusion

The study uncovers the involvement of specific genes (ARG1, DUSP1, F13A1, NFIL3, CCR7, ADM, PTGS2, ID3, FAIM3, HLA-DQB1) and immune cells (neutrophils, T cells, macrophages, dendritic cells) in the pathogenesis of IS, suggesting their importance in understanding and potentially targeting the disease.

## Data Availability

The datasets presented in this study can be found in online repositories. The names of the repository/repositories and accession number(s) can be found in the article/Supplementary material.

## Footnotes

^1^ http://www.ncbi.nlm.nih.gov/geo

^2^ http://metascape.org/gp/index.html

^3^ https://www.string-db.org/

^4^ https://cibersortx.stanford.edu/
